# Unraveling Omnivory and Community Interactions Between Primary Producers and an Apex Predator

**DOI:** 10.1002/ece3.71181

**Published:** 2025-04-07

**Authors:** Ashlee J. Mikkelsen, Andreas Zedrosser, Agnieszka Sergiel, Keith A. Hobson, Nuria Selva, Anne G. Hertel

**Affiliations:** ^1^ Department of Natural Sciences and Environmental Health University of South‐Eastern Norway Borre Vestfold‐Telemark Norway; ^2^ Department of Integrative Biology University of Natural Recourses and Applied Life Sciences Vienna Austria; ^3^ Institute of Nature Conservation Polish Academy of Sciences Krakow Poland; ^4^ Environment and Climate Change Canada Saskatoon Saskatchewan Canada; ^5^ Deparetment of Biology University of Western Ontario London Ontario Canada; ^6^ Estación Biológica de Doñana CSIC Sevilla Spain; ^7^ Department of Biology Ludwig Maximilians University of Munich Planegg‐Martinsried Germany

**Keywords:** brown bear, carnivore, omnivory, primary productivity, stable isotopes, *Ursus arctos*

## Abstract

The effects of climate and plant phenological changes on herbivorous species are widely recognized, yet less research has focused on predatory species, even though vegetative components can account for large proportions of their diet. The historical focus on predation through the lens of simple interactions between obligate carnivores and their prey oversimplifies many species' roles within ecological communities and minimizes other, equally important community functions. We used a long‐term, individual‐based dataset on an omnivorous species, the brown bear ( *Ursus arctos* ), to identify long‐term diet patterns and factors contributing to annual variation in diet. We used carbon and nitrogen stable isotopes measured in hair and Bayesian mixing models to determine annual diet among three demographic classes and then used linear mixed models to relate diet to indices of food availability. Variation in both carbon and nitrogen values were explained by bilberry ( *Vaccinium myrtillus* ) productivity. Additionally, even as the moose population increased over time, there was no increase in the proportion of moose in the diet. The variation in the proportion of moose in the diet slightly decreased throughout the study, while the proportion of bilberry became increasingly more variable. Our results highlight that even though vegetative diet components are typically considered less important to predator ecology, brown bear diet in Sweden responded to changes in berry availability, regardless of prey availability. It will be crucial to put more emphasis on the vegetative parts of diets as we predict how species and ecological communities respond to climate change because predators serve many more functions within their community besides predation alone.

## Graphical abstract

Even though brown bears are typically described as carnivores, our results suggest that their diet is driven by primary production rather than prey availability. We use this species as a model to highlight how plant phenology and climate change may have a stronger impact on omnivores than we currently understand.

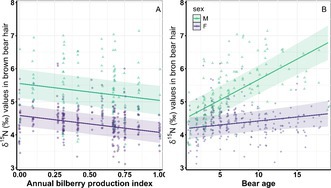

## 1 Introduction

Food webs have traditionally modeled omnivorous species as static consumers exerting constant pressure on multiple resources on distinct trophic levels within stable systems (McLeod and Leroux 2021; Kondoh 2003). However, natural systems are rarely at stable equilibrium, and community interactions are naturally variable (Felicetti et al. 2003; Ushio et al. 2018). For instance, primary productivity varies annually related to climate and insect or pathogen abundance (Bjerke et al. 2014) and organisms can respond to this variation by changing their diet based on resource availability (Deacy et al. 2018; Felicetti et al. 2003). Thus, omnivorous species can rapidly respond to relieve pressure from an exhausted resource while increasing pressure on another, more abundant resource (Kondoh 2003), even if the second resource is a less preferred food (Zhang et al., 2021).

Most mammalian predators eat species from several trophic levels, with few exceptions (Clauss et al. 2010; Yoshimura et al. 2021). Yet historically, predators have been studied through simple interactions between large, charismatic carnivores and their prey, which oversimplifies many omnivores' roles within ecological communities (Miller et al. 2001). This likely overlooks trophic interactions that may be important to community stability (Kratina et al. 2012) and essential to predict community shifts in a changing climate (Gutgesell et al. 2022).

Despite the need for a deeper understanding of omnivore species beyond their roles as predators, measuring all diet components in wild species and complementary responses to environmental change is difficult (Davis and Pineda‐Munoz 2016). One way to estimate diet in wildlife is through stable isotope analysis (Tieszen and Boutton 1989). This method is based on the principle that the ratio of naturally occurring stable isotopes varies across the earth and among different food types, such as C‐4 and C‐3 plants or marine and terrestrial animals, and all organisms must build their tissues from molecules they consume (Tieszen and Boutton 1989). Thus, the stable isotope value of an organism's tissue will most closely resemble that of its dominant foods (Semmens et al. 2009).

In addition to diet estimation, accurate measures of food availability are difficult to obtain, especially over time periods long enough to detect change (Davis and Pineda‐Munoz 2016). Even within the same population of a single species, there will be differences in diet among individuals (Edwards et al. 2011), demographic classes (Beck et al. 2007), across space (Stern et al. 2024), and time (Davis and Pineda‐Munoz 2016), which can obscure general patterns. For example, within a species, different populations may have different responses to changes in specific resources, such as mast crops (Hertel et al. 2019; Schwartz et al. 2010). Thus, determining the effect of variation in resources on the diet of omnivore species is challenging.

We used a dataset on an omnivore mammal, the brown bear ( *Ursus arctos* ), to estimate annual diet proportions of common foods as well as identify diet patterns and drivers over 25 years. We used carbon (*δ* ^13^ *C*) and nitrogen (*δ* ^15^ *N*) stable isotopes measured in hair of known individual bears in south‐central Sweden to estimate annual diet among different demographic classes. We focused on the five primary diet components of bears in this system: ants (*Formnica* spp. and *Camponotus* spp.), bilberry ( *Vaccinium myrtillus* ), crowberry *(Empetrum nigrum)*, lingonberry *(Vaccinium vitus‐vitae)*, and moose (*Alces alces*; Stenset et al. 2016). Berry production in Scandinavia is variable both temporally and spatially (Hertel et al. 2016). Based on previous stable isotope analyses (Mikkelsen et al. 2023), we expected bilberry to make up the greatest proportion of the brown bear diet; however, meat is considered higher quality than berries (Pritchard and Robbins 1990). Thus, while it accounts for a small portion of diet, we expected moose availability to have a disproportionally strong effect on brown bear diet proportions (Hypothesis 1). Specifically, we expected the proportion of moose in the diet to be positively associated with moose availability and the proportion of bilberry to be negatively associated with moose availability. However, diet proportions are estimates from Bayesian mixing models that have limitations, assumptions, and uncertainties (Semmens et al. 2009; Stock et al. 2018). Therefore, we also evaluated variation in the isotope values themselves. *δ* ^15^ *N* values are used as an indicator of trophic level, and within our system, an increase in trophic level (consuming more animal‐derived foods) should correspond to estimated diet changes and have similar relationships with landscape variables. Specifically, we considered that each isotope may be routed through the body differently and reflect different diet patterns (Hypothesis 2; Podlesak and McWilliams, 2006). *δ* ^15^ *N* should be most sensitive to changes in moose availability; *δ* ^15^ *N* values should increase in years with greater moose availability as bears capitalize on the greater availability of a protein and calorie‐dense resource. Meanwhile, berries are abundant in Scandinavian brown bear diet (Mikkelsen et al. 2023) and consist predominantly of carbohydrates and sugars. Thus, we expect *δ* ^13^C values to be most sensitive to changes in bilberry availability, as small changes in carbon from an increase in moose consumption are likely to be overwhelmed by the greater prevalence of carbon in berries.

## 2 Methods

### 2.1 Study System

Our study area encompassed ~13,000 km^2^ in Gävleborg and Dalarna counties in southcentral Sweden with low human density and heavily managed forests of Scots pine ( *Pinus sylvestris* ) and Norway spruce ( *Picea abies* ). Bears were captured via remote drug delivery from a helicopter (Arnemo and Evans 2017) in spring soon after den emergence (March–May). All capture procedures were conducted in accordance with the Swedish Environmental Protection Agency, Swedish Board of Agriculture, and Swedish Ethical Committee on Animal Research.

### 2.2 Sample Collection

Bear hair samples were collected from between the shoulders of brown bears during spring captures 1995–2020. Bears molt annually, beginning in May–June and continuing into October (Jacoby et al. 1999; Jimbo et al. 2020). There is approximately a one‐month lag between a diet change and total equilibrium in hair (Hilderbrand et al. 1996; Felicetti et al. 2003), so a diet change in May would be present in hair cells formed in June. Therefore, hairs represent the accumulated diet over most of the active season in the year prior to capture and collection. After collection, hair samples were placed in individual paper envelopes, labeled accordingly, and stored dry at room temperature. To estimate brown bear diet proportions, we also collected brown bear foods within the study area in 2014 and 2015. We collected hair from between the shoulder blades of local, wild moose harvested during the regular hunting season in 2015. Because the moose harvest in Sweden includes all demographic classes of the population, our moose hair sample set includes adults and sub‐adults of both sexes. Wild berries (fruits) of the three primary species consumed (bilberry, lingonberry and crowberry) were collected from random locations within the study area in summer 2014. Ants of the genera *Formica* and *Camponotus* were also collected within the study area in the summer of 2014. Specimens were mostly adult workers collected from ant hills (*Camponotus*) and by sampling coarse woody debris and tree stumps (*Formica*) in clearcuts of different age classes at random locations in the study area.

### 2.3 Stable Isotope Analysis

When processing hair samples for stable isotope analysis, we separated as much underfur as possible out of the sample and removed large surface contaminants. Preparatory procedures for hair followed the protocol for cortisol concentration measurement (Macbeth et al. 2010; Sergiel et al. 2017; Appendix S2). We washed each sample three times with 40 μL HPLC grade methanol per mg hair for 3 min per wash to remove other external contaminants (Sergiel et al. 2020). After the hair had dried for at least 24 h, it was ground to a fine powder in a mixer mill (Retsch MM4000; Retsch GmbH, Germany) at 30 Hz and then put into plastic vials.

Vegetation and insect samples were washed in distilled water, dried, and powdered prior to stable isotope analyses. 1 mg of the powder from all samples was measured into precombusted capsules. To measure *δ* ^13^ *C* and *δ* ^15^ *N* in hair, we followed Koehler et al. (2019). Powdered samples were combusted at 1030°C in a Carlo Erba NA1500 or Eurovector 3000 elemental analyzer. The resulting N_2_ and CO_2_ were separated chromatographically and introduced to an Elementar Isoprime or a Nu Instruments Horizon isotope ratio mass spectrometer. We used two reference materials to normalize the results to VPDB and AIR: BWBIII keratin (*δ* ^13^ *C* = −20.18, *δ* ^15^ *N* = +14.31 per mil, respectively) and PRCgel (*δ* ^13^ *C* = −13.64, *δ* ^15^ *N* = +5.07 per mil, respectively). Within‐run (*n* = 5) precisions as determined from both reference and sample duplicate analyses and from QA/QC controls were ±0.1 per mil for both *δ* ^13^ *C* and *δ* ^15^ *N*.

We corrected *δ* ^13^C values for the anthropogenic depletion of ^13^C in the atmosphere by applying a −0.022‰ correction per year (Chamberlain et al. 2005) and used results from published feeding experiments on ursids (Felicetti et al. 2003; Hilderbrand et al. 1996; Rode et al. 2016) to estimate the isotopic discrimination factors (TDFs) between bear hair and bear serum (Appendix S1.a). We used a similar procedure to estimate TDFs between moose hair and moose meat and offal (Appendix S1.b).

### 2.4 Statistical Analysis

To answer our hypotheses stated above, we did two separate analyses. First, we used stable isotopes and Bayesian mixing models to estimate annual dietary proportions among three different bear demographic classes (females with dependent offspring, solitary females, and solitary males) to estimate annual dietary proportions of five foods over the 25‐year study period. Because we had an unusually large dataset (almost 700 records) the Bayesian mixing models using the full data size resulted in prohibitively long run times (over 2 weeks and 5 million iterations without conversion). Therefore, when estimating diet proportions, we subset the data by reproductive class and estimated diet for each class separately.

Second, we used linear mixed models to explain annual variation in dietary proportions and stable isotopes relative to indices of annual food availability. Sample size was not an issue for running these models, so all three reproductive classes were included in the same model when modeling variance in isotope values. We tested for trends in diet components and focused on longitudinal trends in the proportions of moose and bilberry in brown bear diet because these were the only foods we had indicators of availability for. Thus, while we cannot account for the availability of all five foods used to estimate brown bear diet, we begin by focusing on the two most prominent foods in Scandinavian brown bear diets.

#### 2.4.1 Dietary Proportion Estimation

Based on previous research, we expected brown bear dietary proportions to vary among the demographic classes of independent (no longer dependent on their mother) bears in our population (Steyaert et al. 2013; Swenson et al. 2007).We subset the stable isotope data by demographic classes (females with dependent offspring, solitary females, and solitary males) and ran three separate diet estimation models using year and bearID as random effects. We used previous diet estimates for our population (Mikkelsen et al. 2023) to derive informative priors for our models. We removed six outliers that had particularly high *δ* ^13^C values and fell outside the mixing polygon. This is standard procedure for mixing models because points outside the mixing polygon cannot be accurately estimated. Points landing outside the mixing polygon also indicate that these consumers were likely eating a food not included in the model. Each model was run with three chains with 3,000,000 iterations, a burn‐in of 1,500,000, and a thin rate of 500. We used graphical output as well as fit statistics to determine if each model had run for a sufficient time to converge and to ensure proper chain mixing (Semmens et al. 2009; Stock et al. 2018). All analysis was completed in R (R Core Team 2024) using package MixSIAR (Stock and Semmens 2013).

Model estimates of the dietary proportions of moose in males had a distinct bimodal distribution, which may arise from the model failing to converge on a single estimate, or from the population having two different diets among males in our sample (i.e., two different possible solutions to the equation). Larger, older males may be more predatory than younger bears (Welch et al. 1997), thus the bimodal distribution may represent the proportion of moose in the diet for subadult males vs. adult males. To test this, we included an additional model for males with an adult and subadult categorical variable as a fixed effect to determine whether this resolved the bimodal distribution.

#### 2.4.2 Annual Variability in Food Availability

**FIGURE 1:**
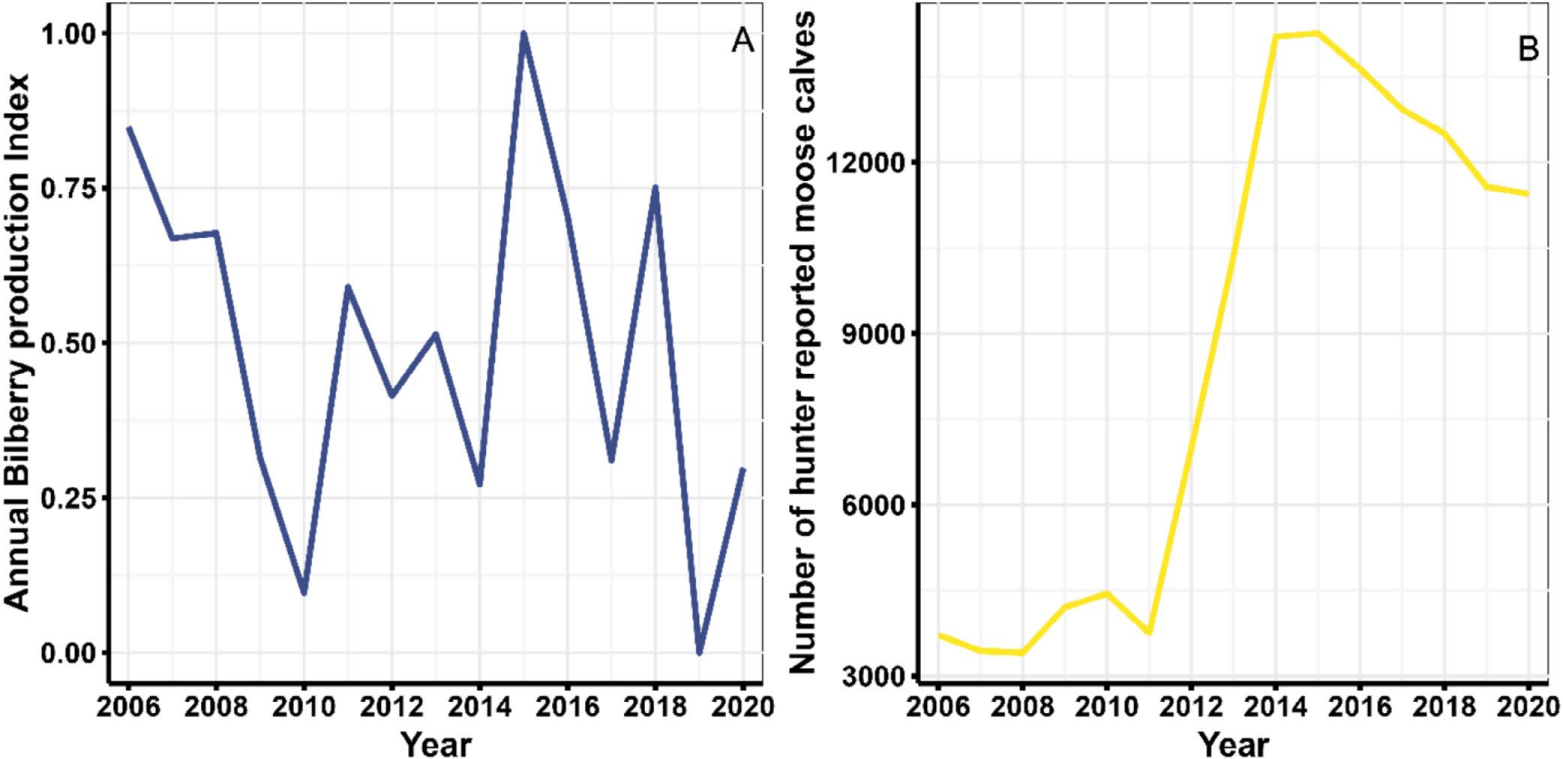
Annual estimates of brown bear food availability for bilberry (A) and moose calves (B) 2006–2020. Bilberry production (A) was estimated from inventory information from the Siljansfors experimental forest in Sweden and a production index < 0.5 indicates fewer berries than average while years with a production index > 0.5 indicated years of above average berry production. Moose calf estimates (B) in Gävleborg and Dalarna counties in Sweden reported in the fall by civilians as part of the nationwide hunter collected data program (Statistik älgdata (https://algdata‐apps.lansstyrelsen.se). This system accounts for surveyor effort, i.e., annual estimates are corrected for observer hours.

**Bilberries**. We used berry inventory information from the Siljansfors Experimental Forest, which is adjacent to the bear monitoring area, to estimate the annual productivity of bilberry. Each year, berry production on 54–60 0.25 m^2^ circular plots was inventoried 2006–2020. The number of ripe bilberries were counted between July and the end of August. Following Hertel et al. (2018), we calculated an annual berry production index as the annual deviation of berry abundance from the 14‐year average for each plot. We then created a model to predict berry production, with year as a fixed effect, and predicted the annual deviation of berries produced. This was scaled between 0 and 1, with indices approaching 0 denoting years of lower‐than‐average berry production and indices approaching 1 denoting years of higher‐than‐average berry production (Figure 1A; Hertel et al. 2018).

**Moose**. Moose harvest and observation data for 2006–2020 was downloaded from Statistik älgdata (https://algdata‐apps.lansstyrelsen.se/algdata‐apps‐stat; Singh et al. 2014) for the counties of Gävleborg and Dalarna. Data in this system are citizen‐reported moose observations in the first 7 days of the hunting season (October) adjusted by observer effort/observation hours. In Sweden, reporting harvested moose is required by law (Singh et al. 2014). The moose observation database also records the sex, age (calves and adults) and the number of calves with an observed female (singles vs. twins), which indicates the overall moose population size, as well as the annual recruitment rate of calves surviving from birth in spring to the fall (Kalén et al. 2022). We used the annual number of total moose observed and harvested 2006–2020 after accounting for hunter effort (Singh et al. 2014) as an indicator of moose population size. We also used the total number of calves observed as an indicator of annual calf production because calves represent the age class most preyed on by bears in the study area (Figure 1B; Swenson et al. 2007).

#### 2.4.3 Linear Mixed Modeling and Model Selection

We used mixed‐effects linear regression models with the lme4 package (Bates et al. 2014) in R (R Core Team 2024) to explain variation and trends in diet proportions, *δ* ^13^ *C*, and *δ* ^15^ *N* values within our population based on a priori hypotheses (Table S2). We used individual ID and demographic class (females with dependent offspring, solitary males, and solitary females) as random effects to explain variation in stable isotope values. We did not include individual ID as a random effect in models explaining variation in dietary proportions because diet was estimated by year and demographic class, not at the individual level, so all members of a reproductive category have the same estimated diet proportion for each of the five foods for each year in the study. We used a build‐up modeling strategy in which we began by determining the best relationship for each covariate considered (linear, log‐linear, or quadratic), and then retained that structure throughout modeling. We used bear age, sex, and annual indices of food availability as fixed effects. All models were compared to the null model to determine whether fixed effects explained more variation than the intercept only, and variables that performed better than the intercept only were used to build more complex models that included additive effects and two‐way interactions. We used an information‐theoretic criterion for small sample sizes (AIC_*c*_) and the relative differences between models (ΔAIC_*c*_) when determining the model with the best fit given the data for final inferences (Burnham and Anderson 2002). For models with similar AIC_*c*_ values, we compared beta estimates, the 95% confidence intervals around the beta estimates, and model variance to select the most parsimonious model (Burnham and Anderson 2002).

Although we had bear data from 1995 to 2020, our annual food availability data was limited to 2006–2020, restricting our inference regarding the drivers of variation in *δ* ^13^C and *δ* ^15^ *N* to this 14‐year period. Prior to analysis, we compared the demographic composition, means, medians, and standard errors of our subset data (2006–2020) to the full dataset (1995–2020) to ensure there were no obvious differences in the data (Supporting Information S3).

In addition to models related to our central hypotheses, we also include 6 a posteriori models (Table S2) that tested for a trend in the variation in the annual estimated proportions of bilberry and moose in brown bear diets. For each demographic category, we calculated a mean proportion of bilberry and moose for all years estimated. Then we used the absolute value of the difference between the annual estimate and the cross‐year mean as an indicator of annual variation:

$$\mathrm{Va}r_{\mathrm{ftd}}=|\mu \left(\mathrm{pro}p_{\mathrm{fd}}\right)-\mathrm{pro}p_{\mathrm{ftd}}|$$

Where *d* is a given demographic class, *f* is a given food source, and *t* is the year.

We used the absolute value of the difference because we were not concerned whether the annual proportion was more than or less than the mean across all years. We then used linear regression analysis with the annual variance as the response variable and year as a continuous variable and demographic class as explanatory variables. We tested for additive effects as well as an interaction between sex and year. The additive only model and the interaction model were compared to the intercept only model using AIC*c* values and model weight (Burnham and Anderson 2002).

## 3 Results

To estimate annual diet proportions over a 25‐year period, 1995–2020, we had a total of 680 records of 278 bears: 190 records from 71 females with dependent offspring (aged 4–24, median number of bears per year = 7, SD = 2.5), 239 records from 118 solitary females (aged 1–21, median number of bears per year = 9.5, SD = 5.0), and 251 records from 120 solitary males (aged 1–29, median number of bears per year = 10, SD = 5.6). Despite the isotopic similarities between two berry species (lingonberry and crowberry) as well as ants and moose, the Bayesian mixing models produced good estimates for these foods (Supporting Information S2, Figure S3). For the linear mixed models explaining variation in diet proportions and isotope values 2006–2020, we had 410 records of 177 bears (aged 1–19); 110 records for females with dependent offspring (aged 4–18), 167 solitary females (aged 1–19), and 133 solitary males (aged 1–19).

### 3.1 Patterns of Diet Composition

**FIGURE 2:**
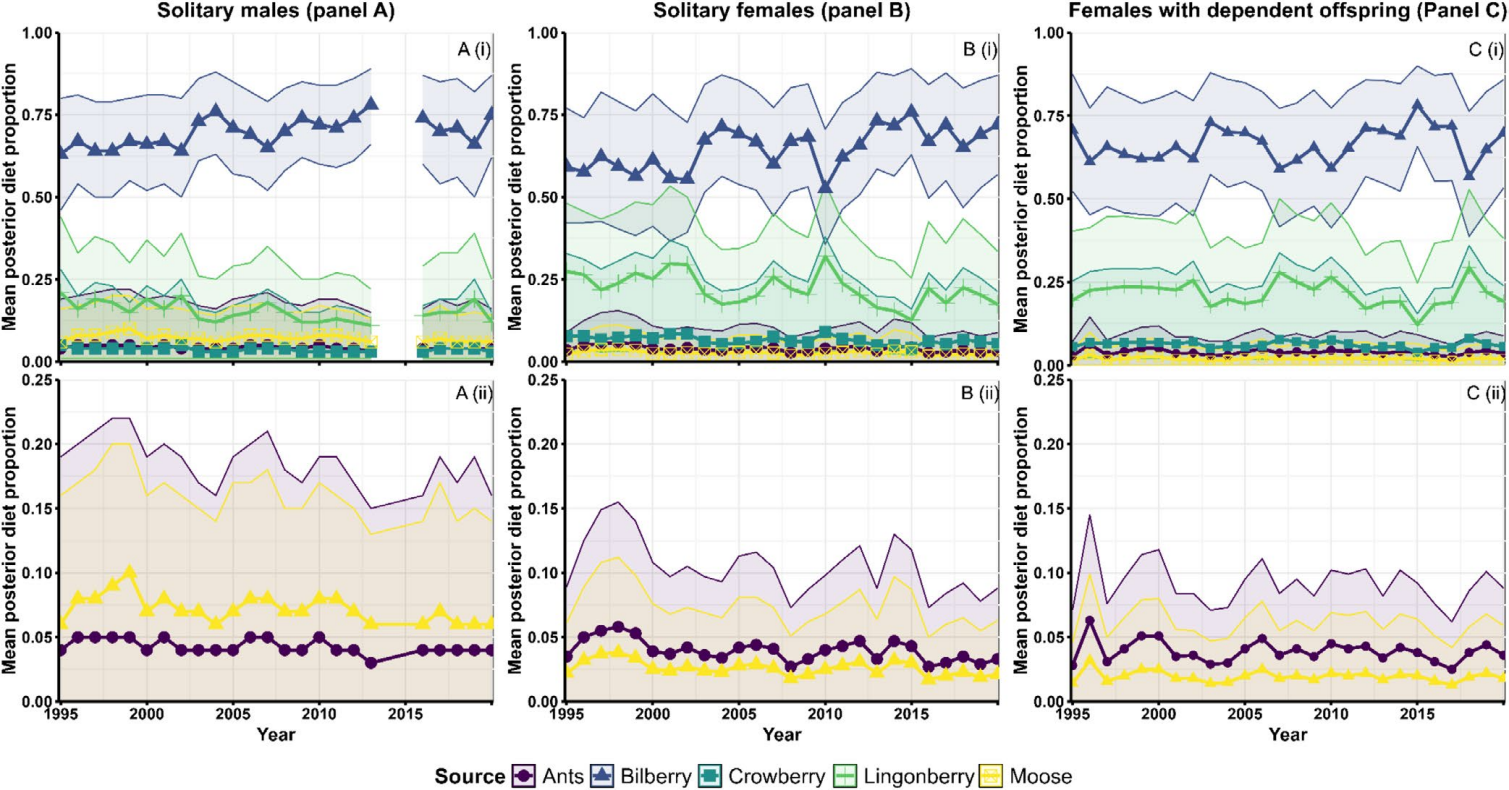
Annual mean estimates of diet proportions for three demographic classes of brown bears in Sweden: Solitary males (A), solitary females (B), and females with dependent offspring (C). Males are included as one demographic class rather than by age group (subadult vs. adult) because sample sizes prevented us from looking at changes in male diet proportions between the two age classes. Panels (i) show proportions of all 5 food sources, whereas panels (ii) only include animal derived foods (moose and ants).

Posterior estimates for the three demographic classes indicated that there was annual variation in brown bear diet proportions, but bilberries made up the greatest proportion of the diet (0.53–0.70) and higher trophic foods (moose and ants) made up small proportions (0.03–0.07; Figure 2) of the diet. For solitary males, moose made up a larger proportion than ants in all years, while the opposite was true for both female classes. Diet among the three demographic classes fluctuated independently; there were no consistent peaks in either moose or ants in a specific year across all classes. There were 2 years in which we had no samples from solitary males (2014 & 2015), so we could not make diet estimates for these years, and thus trends in diet proportions are confounded for solitary males.

Ants and moose were consumed in similar proportions across years (Figure 2 A(ii), B(ii), and c(ii))., but there was evidence for a slight decline in the proportion of moose over time $\mu_{\left(\text{PropMoose}\right)}=\beta_{0}+\beta_{\left(\text{Year}\right)};{\hat{\beta }}_{\left(\text{Moose}.\text{Year}\right)}=-0.02,\mathrm{SE}=0.002$; Table S2); the model that accounted for a trend in diet had all of the model weight (*w*_i_ = 1.00) and the 95% confidence interval did not contain zero. Meanwhile, there was weak evidence that the proportion of bilberry slightly increased over time ($\mu_{\left(\left(\text{PropBilberry}\right)\right)}=\beta_{0}+\beta_{\left(\text{Year}\right)};{\hat{\beta }}_{\left(\text{Bilberry}.\text{Year}\right)}=0.01,\mathrm{SE}=0.005$; Table S2); the intercept only model had considerably more weight than the model that accounted for a linear trend (Intercept *w*_i_ = 0.94) but the 95% confidence interval around the beta estimate did not contain zero. The beta estimates of trends in moose and bilberry translate to changes of less than 1% per year, thus while they have statistical support, their biological relevance is unknown.

The proportion of bilberry in the diet was not correlated with either the bilberry production index or moose availability; the intercept only model had much more support in the data than the model that accounted for bilberry and moose availability ($\mu_{\left(\text{PropBilberry}\right)}=\beta_{0};$ΔAIC_*c*_ = 19.33; Table S2). There was strong evidence for small, negative correlations between the proportion of moose in the diet and both moose availability and bilberry production (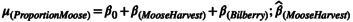 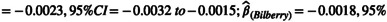 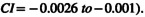 The model explaining variation in the proportion of moose in the diet that accounted for resource availability had more support in the data than the intercept only model (ΔAIC_*c*_ = 32.5; Table S3). Thus, while availability did not seem the affect the proportion of bilberry, it did affect the proportion of moose in the diet; the proportion of moose in the diet decreased with increasing availability of bilberry and decreased in years with greater moose availability.

### 3.2 Patterns in Stable Isotope Values

**FIGURE 3:**
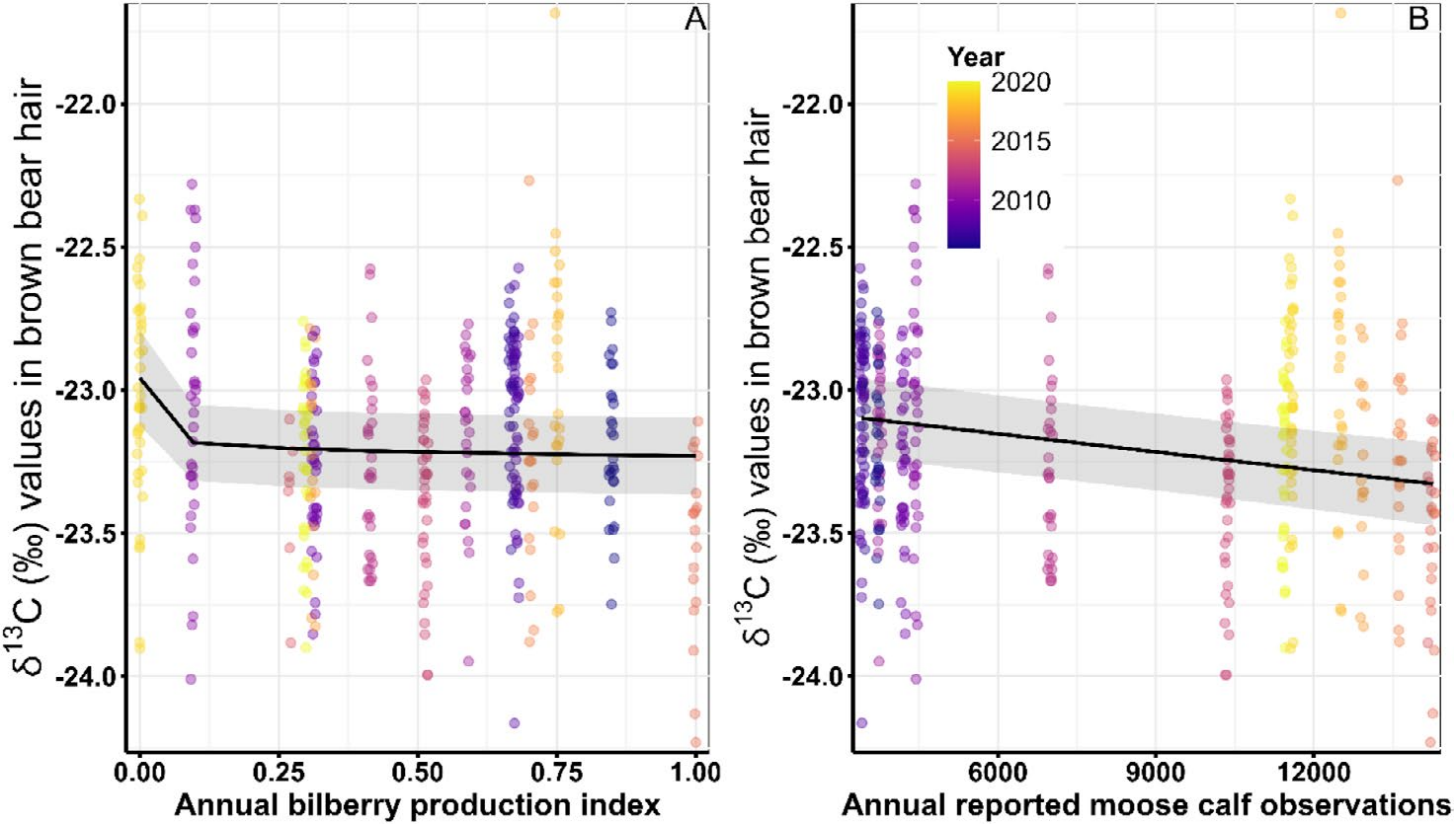
Fitted relationships between *δ* ^13^ *C* measured in brown bear hair collected in Southcentral Sweden 2006–2020 and the annual index of bilberry production (3A) and annual number of estimated moose calves from hunter observations after accounting for observer effort (3B). Lines are the fitted relationships from the top model with 95% confidence intervals shaded around the lines. Colored points represent the raw data collected, with darker colors representing earlier years and lighter colors representing later years.

Among foods included in the diet analysis, moose and ants had the highest *δ* ^13^C values, crowberry and lingonberry had median values, and bilberry had the lowest *δ* ^13^C values. Variation in brown bear *δ* ^13^C values was best described by a log‐linear relationship with bilberry production and the number of moose calves observed in the year in which hair was grown ($\mu_{\left(\delta^{13}C\right)}=\beta_{0}+\beta_{\left(\text{Bilberry}\right)}+\beta_{\left(\text{Calves}\right)}$; Table S2). Brown bear *δ* ^13^C values were lower (more similar to bilberries) in years with greater bilberry production (${\hat{\beta }}_{\left(\ln\left(\text{Bilberry}\right)\right)}=-0.21,95\%\mathrm{CI}=-0.30\;\text{to}-0.12$). They were also lower (more similar to bilberry) in years with greater numbers of observed moose calves (${\hat{\beta }}_{\left(\text{MooseCalves}\right)}=-0.25,95\%\mathrm{CI}=-0.35\;\text{to}-0.14$). Though well supported, the relationship between *δ* ^13^C values and bilberry was small (Figure 3A). We evaluated the strength of the effect of bilberry by removing it from the model and comparing the resulting model performance metrics to the model that included the effect of bilberry. The model that accounted for bilberry production had a ΔAIC_*c*_ 14.1 units lower and a model weight over 1000× greater (0.9979 vs. 0.0009) than the model without the effect of bilberry production. The high statistical significance but small beta estimate may be related to the overall small difference among *δ* ^13^ *C* values in the foods included in this study.

**FIGURE 4:**
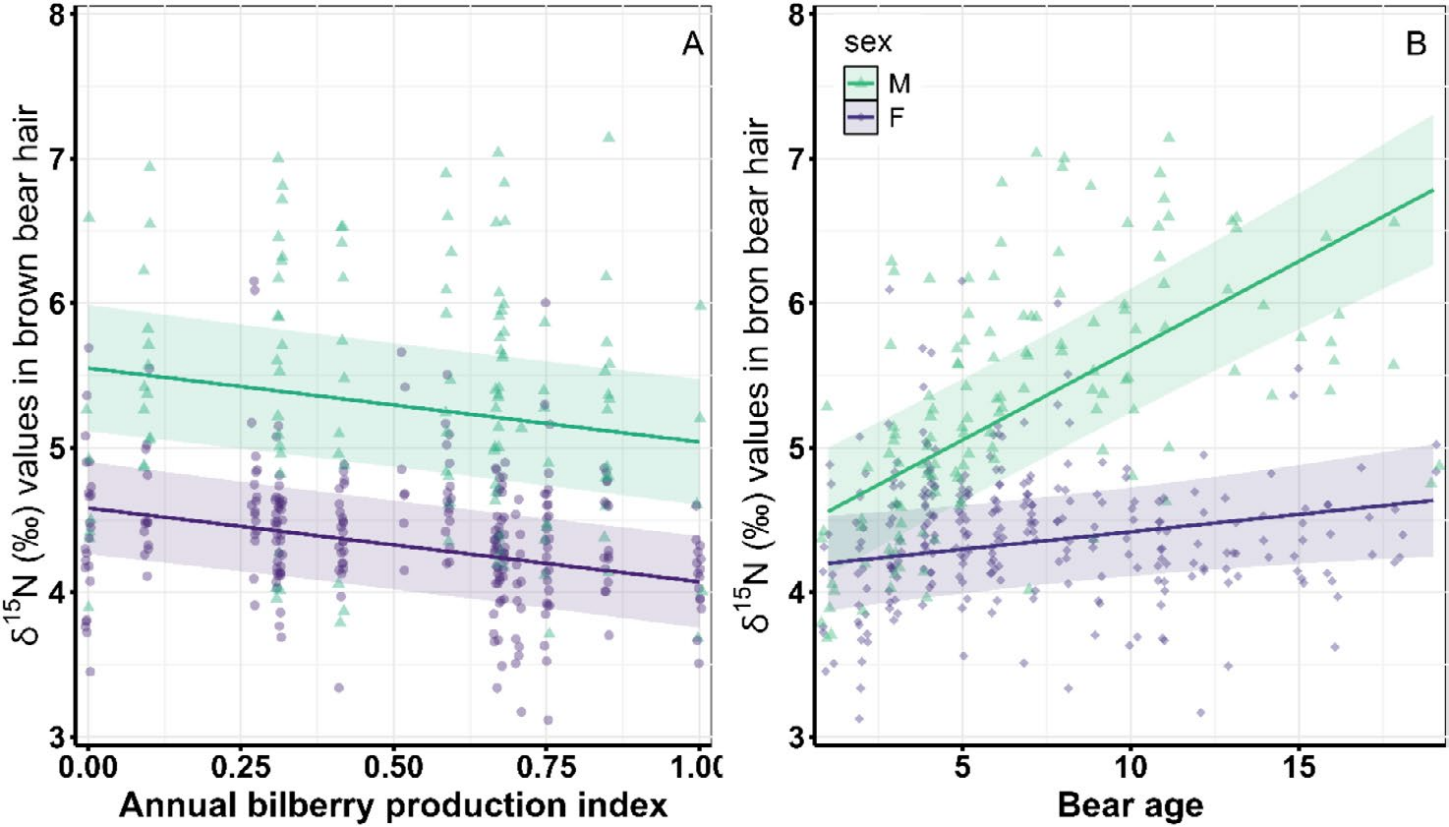
Fitted relationships between *δ* ^15^ *N* measured in brown bear hair collected in Southcentral Sweden 2006–2020 and the annual index of bilberry production (A) and bear age and sex (B). There was no difference between solitary females and females with dependent offspring, so these were combined into a single “female” category. Lines are the fitted relationships from the top model with 95% confidence intervals shaded around the lines. Colored points represent the raw data colored by sex.

*δ* ^15^ *N* were highest in moose and ants, followed by bilberry, lingonberry, and crowberry had the lowest *δ* ^15^ *N* values. Variation in *δ* ^15^ *N* was best described (*w*_*i*_ = 0.9) by the model that accounted for bilberry production with a one‐year time lag (berry production in *t* affecting stable isotope values in *t + 1*), bear age and sex, and an interaction between age and sex ($\mu_{\left(\delta^{15}N\right)}=\beta_{0}+\beta_{\left(\mathrm{Age}\right)}+\beta_{\left(\mathrm{Sex}\right)}+\beta_{\left(\text{Bilberr}y_{\left(\text{lagged}\right)}\right)}+\beta_{\left(\mathrm{Age}*\mathrm{Sex}\right)}$; Table S2, Figure 4A,B). The next best model was identical, except it also accounted for the number of moose harvested, but the effect of moose had little to no support; the moose model had less support in the data (ΔAIC_*c*_ = 4.3, w_*i*_ = 0.1) and the beta estimate for moose overlapped zero by 13% (Figure S4).

*δ* ^15^ *N* values were lower (more similar to bilberry values) in years with greater bilberry production (${\hat{\beta }}_{\text{Bilberry}}=-0.18,95\%\mathrm{CI}=-0.24\;\text{to}-0.12$) and *δ* ^15^N values increased with bear age, but the effect differed between the sexes (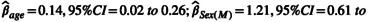 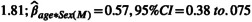).

### 3.3 Trends in Variance of Diet

**FIGURE 5:**
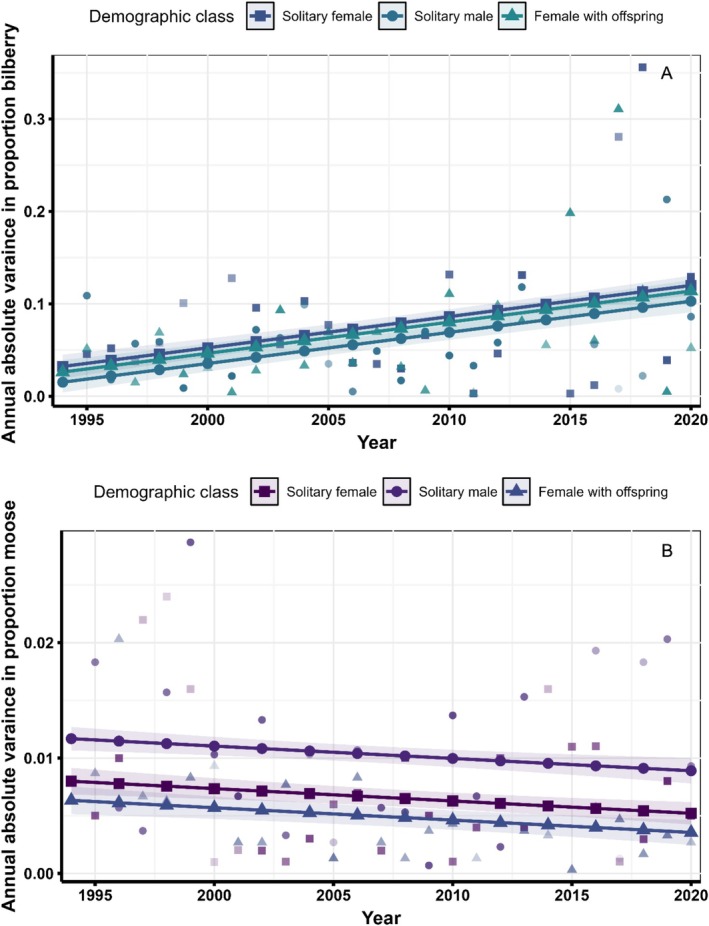
Trends in the variance of two diet components estimated from stable isotopes in brown bear hair from the Scandinavian population (1995–2020). For each demographic class, we calculated dietary variance as the absolute difference in estimated diet proportions in year *t* and Demographic class *d* from the overall mean of demographic class *d*. Lines represent the results from the top model of trends in variance through time for each demographic class while points represent the observed absolute variance for each demographic class.

A posteriori models of the variance of the dietary proportions of bilberry and moose revealed strong support for trends in both food sources (Figure 5 and Table S2). Throughout the study, variance in the annual proportion of bilberry in the diet increased by more than three times the starting variance (${\hat{\beta }}_{\left(\text{Year}.\text{Bilberry}\right)}=$ 0.003, 95%CI = 0.002–0.004). Unlike bilberry, the annual variation in moose declined over time (${\hat{\beta }}_{\left(\text{Year}.\text{Moose}\right)}$= −0.0001, 95%CI = −0.00016 to −0.00004). The beta estimates around moose variation are very small because (1) moose constituted a small proportion of brown bear diets and (2) varied little among years. However, there is still evidence that variation in moose declined.

## 4 Discussion

Predator population dynamics, behavior, and diet are typically associated with changes in prey populations, which means that predation and prey interactions tend to be the primary concerns when designing conservation and management programs or even understanding the general ecology of predatory species. Our results suggest that the opposite can be true—even for a large apex predator. Brown bear diet changed in response to berry productivity, regardless of moose availability, indicating that landscape changes that affect berry production will have larger impacts on bears than changes in prey populations. Both moose calves and berries are temporarily available food resources (Stenset et al. 2016), but berries are available for a longer period and are the primary food source during hyperphagia (Deacy et al., 2018; Hertel et al. 2018). Thus, based on the overwhelming contribution of berries to bear diet (Mikkelsen et al. 2023) it should not be surprising that berry availability has a stronger influence on brown bear diet than moose availability.

Brown bear hair *δ* ^15^ *N* values were closely related to the proportion of moose in the diet, but neither *δ* ^15^ *N* values nor estimated diet proportions of moose were related to indices of moose availability. Rather, both *δ* ^15^ *N* values and dietary proportions of moose were explained by bilberry availability. Meanwhile, the dietary proportion of bilberry was unrelated to either moose or bilberry availability. Variation in *δ* ^13^ *C* values was also explained by bilberry availability, though some variation in *δ* ^13^ *C* was also explained by annual moose calf production.

The unintuitive relationship in which *δ* ^13^ *C* values are more similar to plant‐derived foods in years with high moose calf observations and more similar to animal‐derived foods during years of low moose observations may be an artifact of timing. Bears tend to predate moose neonates in the spring soon after calving (Swenson et al. 2007), while calf observations are reported by the public in October. Brown bears can be a substantial source of mortality for moose calves in Sweden (~25%; Swenson et al. 2007), and so this relationship may arise from bears predating (and eating) more moose calves in the spring, resulting in fewer calves to be counted in the fall. However, due to the overall size of the moose population (Jensen et al., 2020) we are uncertain whether bear predation could have this strong of an effect on reported moose numbers.

Prior to 2011, annual moose calves in the study area fluctuated around 3000–4000, then rapidly increased until numbers restabilized in 2014 around 13,000 (Statistik älgdata). As a species with a diverse diet, we expect brown bear to rapidly respond to changes in resource availability, particularly a preferred food source like moose (Deacy et al., 2018; Felicetti et al. 2003). Yet, the proportion of moose in the diet did not increase with calf observations; rather, it appears to be stable or decrease through time. This may be a result of the brief temporal window in which moose calves are vulnerable to brown bear predation (Swenson et al. 2007), and even at the lowest calf numbers documented in this study, the bear population may have been saturated and could not consume any more moose (Charnov 1976). The variability associated with the proportion of moose declined through time, which may also be related to diet saturation. If bears are saturated by the number of moose calves on the landscape, then we expect the proportion of moose in the diet to be dictated by handling time alone, which is likely less variable than both search and handling time.

Because brown bears did not change their diet in response to increasing moose availability, it may indicate that brown bears did not evolve to be dependent on animal‐derived foods. In aquatic systems, predators often have a stronger preference for higher‐trophic foods but get most of their energy from an abundance of lower trophic foods (Zheng et al. 2021). This may also be true for terrestrial systems. While bears may prefer higher trophic foods when given a free choice with no searching or handling times, they likely evolved feeding heavily on lower trophic foods and exploiting higher trophic foods less often (Robbins et al. 2022; Rode et al. 2021).

As climate continues to change in the arctic, bears may become less dependent on moose calves. Areas in and near the arctic are projected to be much warmer with a longer growing season (Box et al. 2019; Inouye 2022), and brown bear's trophic position tends to decrease with greater primary productivity (Albrecht et al. 2024; Bojarska and Selva 2012). This effect may be particularly strong if lower trophic foods become available earlier in the season and overlap with moose calving, incentivizing bears to spend less time on calf predation for more time foraging on plant‐based foods, as has been seen in North America (Deacy et al., 2018). However, community responses to climate change are complex and difficult to predict, and there was a large increase in the variance of the proportion of bilberry in the diet through time. Whether this is an indicator of bears switching to an alternative food source not considered in this analysis or an indication of bilberry availability becoming increasingly variable annually requires further study.

It must be noted that this study considers a simplified subset of the larger community by focusing on only a few interactions (brown bears, moose, ants, and 3 berry species) and attempts to place it within the larger framework of foraging and community ecology theories. We used five foods that have previously been identified as important for bears in Scandinavia (Mikkelsen et al. 2023; Stenset et al. 2016) to estimate diet, but we may have inadvertently excluded foods such as domestic oats ( *Avena sativa* ) that could contribute to variation in brown bear diet. Further, we only had data on the annual availability of two food sources, so this analysis cannot account for other landscape availability factors that may contribute to changes in brown bear diet. Additionally, we only collected food samples during a single year; thus, we cannot account for annual variation in isotopic values of the food sources we used to estimate bear diet. For instance, we applied a correction to account for the Suess effect in bear samples (Chamberlain et al. 2005), but this same phenomenon would also affect the ^13^C values of all members within the community. Therefore, the slight decline in the proportion of moose and ants could be attributed to the assumption that food samples collected in 2014 and 2015 were representative of each year within the study.

However, our results add to the growing body of evidence that meat may be overemphasized in the diets of some omnivorous species (Deacy et al. 2017; Robbins et al. 2022; Rode et al. 2021), which minimizes the other roles these species play, such as nutrient cyclers and seed dispersers (Borchert and Tyler 2011; Harrer and Levi 2018; Reimchen 2017). Beyond ursid species, modern monitoring tools, such as DNA metabarcoding, camera traps, and stable isotope analysis, have documented “novel” foraging behaviors in a wide variety of predators, such as bonnethead sharks ( *Sphyrna tiburo* ) eating seagrasses (Leigh et al. 2018), crocodilians (order Crocodilia) eating fruit and dispersing seeds (Platt et al. 2013), or wolves ( *Canis lupus* ) foraging on berries (Homkes et al. 2020).

It is unclear how many other omnivorous species are sensitive to changes in primary production. While much work has been done on trophic cascades via top‐down effects, more research is needed on trophic transcendence via bottom‐up effects. For example, within Scandinavia, annual weather patterns explain little variation in annual berry production (Hertel et al. 2018; Selås 2000). This is because fruit production may be negatively affected by short but extreme weather events or a complex interaction of conditions over the full growing season (Orsenigo et al. 2014). Additionally, human modifications to the landscape, such as the conversion of wildlands to agriculture or timber production, change the structure of ecological communities and can interact with climate change to exacerbate changes in resource availability (Pirotta et al. 2022). Ultimately, omnivorous species may be doubly affected by changes in plant phenology and productivity, once through a direct change in plant‐based resource availability as a food source (Deacy et al. 2017; Hertel et al. 2018), then again as prey species also respond to changes in primary production (Kobayashi et al. 2012).

## Author Contributions

**Ashlee J. Mikkelsen:** conceptualization (lead), formal analysis (lead), visualization (lead), writing – original draft (lead). **Andreas Zedrosser:** conceptualization (equal), data curation (equal), formal analysis (equal), funding acquisition (lead), project administration (lead), writing – original draft (equal), writing – review and editing (equal). **Agnieszka Sergiel:** data curation (equal), investigation (equal), methodology (equal), writing – original draft (equal), writing – review and editing (equal). **Keith A. Hobson:** data curation (equal), formal analysis (equal), methodology (equal), validation (equal). **Nuria Selva:** conceptualization (equal), data curation (equal), funding acquisition (equal), writing – review and editing (equal). **Anne G. Hertel:** conceptualization (equal), formal analysis (equal), funding acquisition (equal), project administration (supporting), supervision (equal), validation (equal), writing – original draft (equal), writing – review and editing (equal).

## Conflicts of Interest

The authors declare no conflicts of interest.

## Supporting information


Data S1.

## Data Availability

Once the manuscript is accepted, we will make the data and all code used in our analyses publicly available in accordance with the policies of the Norwegian government and the University of South‐eastern Norway (https://bibliotek.usn.no/publishing/open‐access/open‐access‐policy/). Data and code will be stored on the USN Research Data Archive. No novel code was used in this analysis.
