# Supplementary material for: Unraveling Omnivory and Community Interactions Between Primary Producers and an Apex Predator
Source: Ecol Evol. 2025 Apr 7;15(4):e71181. doi: 10.1002/ece3.71181 (PMC11974456; doi:10.1002/ece3.71181)
Supplement: Supplementary file 1 — Data S1. [file ECE3-15-e71181-s001.docx]

**Unraveling dynamic omnivory and community interactions between primary producers and an apex predator**

## **Supplementary materials**

# S.1 Stable isotope corrections and TDF estimations

#### S.1.1 Bear hair stable isotope corrections

To estimate the isotopic discrimination factors (TDFs) between bear hair (which we sampled) and blood plasma (which is the most commonly measured medium to detect changes between diet and isotope values in short-term feeding experiments), we used results published from three controlled feeding experiments on brown bears (*Ursus arctos*), American black bears (*U. americanus*), and polar bears (*U. maritimus*) that estimated the difference between the stable isotope content of the diet and the stable isotope content of various tissues to develop linear relationships for predicting the isotope values of a tissue based on diet (Felicetti et al., 2003; Hilderbrand et al., 1996; Rode et al., 2016). The isotopic values of plasma and hair are similar in bears raised on a constant diet (Hilderbrand et al., 1996), thus, we assumed that our isotope values from hair were approximately equivalent to plasma isotope values. We combined the results from Felicetti et al. (2003) and Hilderbrand et al. (1996) by taking the mean of both intercepts and slopes to derive intermediate linear equations:

$$\delta^{13}C_{plasma}= -10.6+0.42 (\delta^{13}C_{food source})$$

$$\delta^{15}N_{plasma}= 5.02+0.90 (\delta^{15}N_{food source})$$

to calculate the expected isotopic plasma values for a brown bear consuming 100% of each food source in our system. We then subtracted the mean isotopic value of each source from the expected isotopic value of a bear consuming 100% of that source (Table S1). For example, a bear in our system consuming 100% moose would have an expected isotopic value for *δ*^15^N_bear hair_ = 6.9‰, while our sample mean *δ*^15^N_moose meat_ = 2.1‰, the difference between these yields a nitrogen TDF for moose of 4.6‰ ($\delta^{15}N_{bear}- \mu\delta^{15}N_{moose}= TDF_{moose}^{\delta^{15}N}$).

#### S.1.2 Moose hair stable isotope corrections

The same laboratory procedures were used for moose hair as were used for bear hair.

We derived source-specific trophic discrimination factors to convert prey meat *δ*^13^C and *δ*^15^N values to hair equivalent values. Because moose in our system were represented by hair samples, we first determined the TDF between moose diet and moose meat and hair. Feeding experiments are rare in wildlife species, particularly for large species. We used published results of controlled feeding experiments on bison (*Bison bison*), elk (*Cervus elaphus*), caribou (*Rangifer tarandus*), and moose (*Alces alces*) to estimate the isotopic discrimination between ungulate hair and muscle under equilibrium conditions (Drucker et al., 2010; Milakovic & Parker, 2013; Rioux et al., 2020; Tieszen & Boutton, 1989). Values of *δ*^13^C between hair and blood or muscle ranged from 0.99‰ in elk to 1.01‰ in bison and *δ*^15^N ranged from 0.77‰ in elk to 1.0‰ in bison. We determined the mean ratio of *δ*^13^C and *δ*^15^N between hair and muscle across published data from wild ungulate species and found that:

$$\mu_{\left( \delta^{13}C \right)}\left( \frac{hair}{meat} \right)=0.99$$

$$\delta^{13}C_{\left( moose hair \right)}=0.99*\delta^{13}C_{\left( moose meat \right)}$$

$\mu_{\left( \delta^{15}N \right)}\left( \frac{hair}{meat} \right)=0.88$

$$\delta^{15}N_{\left( moose hair \right)}=0.88*\delta^{15}N_{\left( moose meat \right)}$$

We used these equations to calculate the expected *δ*^13^C and *δ*^15^N of moose meat from sampled moose hair (Caut et al., 2008; Kurle et al., 2014).

# S.2 Isotopic similarity among foods

Despite their isotopic similarities (Figure S2), lingonberry (*Vaccinium vitus-vitae*) and crowberry (*Empetrum nigrum*) had a low negative correlation (Figure S3), which strengthens our confidence in the posterior diet estimates and the model’s ability to differentiate between these food sources. Moose and ants (genera *Formica* and *Camponotus*), like lingonberry and crowberry, are also isotopically similar (Figure S2), yet the posterior distributions are unimodal and narrow, indicating good distinction between the two sources (Phillips et al., 2005).

# S.3. Oat bears

Some bears in this population are known to frequent agricultural fields to eat domestic oats (*Avena sativa*), but we did not include oats in our analysis or mixing polygon. Based on previous studies that used GPS locations to detect this behavior (Steyaert et al., 2016), our records included 80 instances of bears known to visit oat fields. Domestic plant species, such as oats tend to have higher *δ*^13^C values than wild plants, thus bears with very high *δ*^13^C values may have been consuming oats, which we cannot account for. Visual checks and linear regression modelling indicated there is a difference in *δ*^13^C values between oat bears and other bears. However, while statistically different, the mean *δ*^13^C value of oat bears was only 6% lower than other bears (beta estimate difference = 0.17, overall distribution of *δ*^13^C = -24.2 to -21.7). Thus, we do not believe that 10% of bears in our data, which have *δ*^13^C values on average 6% lower than the rest of the sample, will skew our results and inferences.

# S.4 Comparison between full dataset and subset of data (limited by food availability data)

Even though this analysis has stable isotope data on individuals bears from 1995–2020, data on berry production and moose populations is limited to 2006–2020. In order to make broad generalizations about landscape influences on diet in this bear population, we show that models results apply to the larger dataset, and not just the 2006–2020 subset. We first used visual checks of sample distributions to ensure there was no obvious bias or trends in sampling across the study (such as sample size slowly increasing with effort as the study proceeded).

Year 2014 represents an odd break-point, in which prior to 2014, there is an even distribution of samples across the three reproductive categories, but after 2014 there are few males in the sample. Males tend to have higher *δ*^15^N values than females, but *δ*^15^N values in two years in which no solitary males were sampled (2014 & 2015) are not abnormally low or at risk of biasing the analysis. Overall mean *δ*^15^N is 4.75 ± 0.03, while in 2015 mean *δ*^15^N = 4.75 and 2014 mean *δ*^15^N = 4.84. The mean *δ*^15^N for 2014 and 2015 are two standard deviations of the global mean, but it's *higher* than the overall mean, rather than *lower* as we might expect in years with no males in the sample.

When we compare the distribution of isotope values of the full data set, and the sub-set data, there is very little difference between them. For *δ*^15^N values, $\mu\delta^{15}N_{\left( Full \right)}=4.75\pm0.03, \mu\delta^{15}N_{\left( 2006-2020 \right)}=4.72 \pm0.04$. For *δ*^13^C values, $\mu\delta^{15}N_{\left( Full \right)}=-23. 14\pm0.34 , \mu\delta^{15}N_{\left( 2006-2020 \right)}=-23.18 \pm0.35$. The distributions of important explanatory variables (year, reproductive category, and age) do not vary across the two datasets. Thus, we are confident that the landscape variables we determined to be important in explaining variation in the isotope values for the latter half of our data (61% of records and 58% of years in the subset, are also likely to be important to the earlier half of the data set, and the data as a whole.

# Literature Cited

Caut, S., Angulo, E., & Courchamp, F. (2008). Discrimination factors (*δ*^15^N and *δ*^13^C) in an omnivorous consumer: effect of diet isotopic ratio. *Functional Ecology*, *22*(2), 255–263. https://doi.org/10.1111/j.1365-2435.2007.01360.x

Drucker, D. G., Hobson, K. A., Ouellet, J.-P., & Courtois, R. (2010). Influence of forage preferences and habitat use on 13 C and 15 N abundance in wild caribou (*Rangifer tarandus*) and moose (*Alces alces*) from Canada. *Isotopes in Environmental and Health Studies*, *46*(1), 107–121. https://doi.org/10.1080/10256010903388410

Felicetti, L. A., Schwartz, C. C., Rye, R. O., Haroldson, M. A., Gunther, K. A., Phillips, D. L., & Robbins, C. T. (2003). Use of sulfur and nitrogen stable isotopes to determine the importance of whitebark pine nuts to Yellowstone grizzly bears. *Canadian Journal of Zoology*, *81*(5), 763–770. https://doi.org/10.1139/z03-054

Hilderbrand, G. V., Farley, S. D., Robbins, C. T., Hanley, T. A., Titus, K., & Servheen, C. (1996). Use of stable isotopes to determine diets of living and extinct bears. *Canadian Journal of Zoology*, *74*(11), 2080–2088. https://doi.org/10.1139/z96-236

Kurle, C. M., Koch, P. L., Tershy, B. R., & Croll, D. A. (2014). The effects of sex, tissue type, and dietary components on stable isotope discrimination factors (*δ*^13^C and *δ*^15^N) in mammalian omnivores. *Isotopes in Environmental and Health Studies*, *50*(3), 307–321. https://doi.org/10.1080/10256016.2014.908872

Milakovic, B., & Parker, K. L. (2013). Quantifying carnivory by grizzly bears in a multi-ungulate system. *Journal of Wildlife Management*, *77*(1), 39–47. https://doi.org/10.1002/jwmg.434

Phillips, D. L., Newsome, S. D., & Gregg, J. W. (2005). Combining sources in stable isotope mixing models: Alternative methods. *Oecologia*, *144*(4), 520–527. https://doi.org/10.1007/s00442-004-1816-8

Rioux, È., Pelletier, F., & St-Laurent, M. H. (2020). From diet to hair and blood: Empirical estimation of discrimination factors for C and N stable isotopes in five terrestrial mammals. *Journal of Mammalogy*, *101*(5), 1332–1344. https://doi.org/10.1093/jmammal/gyaa108

Rode, K. D., Stricker, C. A., Erlenbach, J. A., Robbins, C. T., Cherry, S. G., Newsome, S. D., Cutting, A., Jensen, S., Stenhouse, G. B., Brooks, M., Hash, A., & Nicassio, N. (2016). Isotopic incorporation and the effects of fasting and dietary lipid content on isotopic discrimination in large carnivorous mammals. *Physiological and Biochemical Zoology*, *89*(3), 182–197. https://doi.org/10.1086/686490

Steyaert, S. M. J. G., Zedrosser, A., Elfström, M., Ordiz, A., Leclerc, M., Frank, S. C., Kindberg, J., Støen, O. G., Brunberg, S., & Swenson, J. E. (2016). Ecological implications from spatial patterns in human-caused brown bear mortality. *Wildlife Biology*, *22*(4), 144–152. https://doi.org/10.2981/wlb.00165

Tieszen, L. L., & Boutton, T. W. (1989). *Stable Carbon Isotopes in Terrestrial Ecosystem Research* (pp. 167–195). https://doi.org/10.1007/978-1-4612-3498-2_11

# Tables and Figures

Table S1. Diet-hair trophic discrimination factors (mean ± SD in ‰) of Δ^13^C and Δ^15^N for brown bear (*Ursus arctos*) calculated using a combination of linear equations from Hilderbrand et al. (1996), Felicetti et al. (1993), and published data from Rode et al. (2016). Trophic discrimination factors calculated using Hilderbrand’s and Felicetti’s linear equation are also included.

|  | This Study | | Hilderbrand et al. 1996 | | Felicetti et al. 2003 | |
| --- | --- | --- | --- | --- | --- | --- |
| Food-source | Δ^13^C | Δ^15^N | Δ^13^C | Δ^15^N | Δ^13^C | Δ^15^N |
| Ants | +4.6 ± 1 | +4.9 ± 1 | +4.9 ± 1 | +4.6 ± 1 | +4.4 ± 1 | +5.1 ± 1 |
| Bilberry | +6.6 ± 1 | +5.3 ± 1 | +6.9 ± 1 | +5.0 ± 1 | +6.4 ± 1 | +5.6 ± 1 |
| Crowberry | +5.1 ± 1 | + 5.6 ± 1 | +5.3 ± 1 | + 5.3 ± 1 | +4.8 ± 1 | + 6.0 ± 1 |
| Lingonberry | +5.2 ± 1 | +5.5 ± 1 | +5.5 ± 1 | +5.1 ± 1 | +4.9 ± 1 | +5.8 ± 1 |
| Moose | +4.6 ± 1 | +4.9 ± 1 | +5.1 ± 1 | +4.6 ± 1 | +4.6± 1 | +5.1 ± 1 |

Table S2. Full list of models considered to explain variation in stable isotope values measured in brown bear hair from an individually marked population in south-central Sweden 1995–2020. Relationships considered were linear, log-linear (ln), and quadratic (^2^) with either no time lag (food availability correlated with the stable isotopes in the same year hair was grown) or with a 1-year time lag (food availability in year *t-1* correlated with stable isotopes in hair grown in year *t*).

| Isotope | Time Structure | Model | ΔAIC*_c_* | w*_i_* |
| --- | --- | --- | --- | --- |
| ***δ*^15^N** | **No time lag** |  |  |  |
|  |  | Age*sex | 0 | 1.0 |
|  |  | Age+sex | 30.2 | 0.0 |
|  |  | Proportion moose in diet | 30.4 | 0.0 |
|  |  | Age | 32.6 | 0.0 |
|  |  | Proportion ants in diet | 44.5 | 0.0 |
|  |  | Sex | 78.5 | 0.0 |
|  |  | Intercept only | 80.2 | 0.0 |
|  |  | (Moose observations)^2^ | 83.4 | 0.0 |
|  |  | Moose observations | 84.4 | 0.0 |
|  |  | Moose calves | 85.0 | 0.0 |
|  |  | Year | 61.2 | 0.0 |
|  |  | ln(Moose observations) | 85.7 | 0.0 |
|  |  | ln(Moose calves) | 85.9 | 0.0 |
|  |  | ln(Bilberry) | 86.0 | 0.0 |
|  |  | (Moose harvest)^2^ | 86.1 | 0.0 |
|  |  | (Moose calves)^2^ | 86.4 | 0.0 |
|  |  | (Moose harvest) | 86.8 | 0.0 |
|  |  | ln(Moose harvest) | 86.8 | 0.0 |
|  |  | (Bilberry)^2^ | 86.9 | 0.0 |
|  |  | Bilberry | 87.1 | 0.0 |
|  |  |  |  |  |
| ***δ*^15^N** | **1-year lag** |  |  |  |
|  |  | Age*Sex+Bilberry^a^ | 0 | 0.95 |
|  |  | Age*Sex+Bilberry+Moose harvest | 6.0 | 0.05 |
|  |  | Age*Sex | 24.4 | 0.00 |
|  |  | Age*Sex+Moose harvest | 31.0 | 0.00 |
|  |  | Age+Sex | 50.4 | 0.00 |
|  |  | Age | 53.1 | 0.00 |
|  |  | Bilberry | 79.2 | 0.00 |
|  |  | (Bilberry)^2^ | 82.6 | 0.00 |
|  |  | Year | 83.9 | 0.00 |
|  |  | (Moose harvest)^2^ | 95.3 | 0.00 |
|  |  | ln(Moose harvest) | 97.2 | 0.00 |
|  |  | Moose harvest | 97.5 | 0.00 |
|  |  | (Moose observations)^2^ | 97.8 | 0.00 |
|  |  | Sex | 99.0 | 0.00 |
|  |  | Intercept | 100.8 | 0.00 |
|  |  | ln(Bilberry) | 102.1 | 0.00 |
|  |  | Moose Observations | 102.4 | 0.00 |
|  |  | ln(Moose observations) | 104.4 | 0.00 |
|  |  | ln(Moose calves) | 104.8 | 0.00 |
|  |  | MooseCalves | 106.5 | 0.00 |
|  |  | (Moose calves)^2^ | 107.70 | 0.00 |
|  |  |  |  |  |
| ***δ*^13^C** | **No time lag** |  |  |  |
|  |  | Year^b^ |  |  |
|  |  | ln(Bilberry)+Moose calves^c^ | 0 | 1.00 |
|  |  | Moose calves | 14.1 | 0.00 |
|  |  | (Moose harvest)^2^ | 14.2 | 0.00 |
|  |  | ln(Moose calves) | 14.7 | 0.00 |
|  |  | ln(Bilberry) | 15.1 | 0.00 |
|  |  | Moose harvest | 15.5 | 0.00 |
|  |  | (Moose calves)^2^ | 15.5 | 0.00 |
|  |  | ln(Moose harvest) | 15.6 | 0.00 |
|  |  | ln(Moose Observations) | 16.8 | 0.00 |
|  |  | Moose Observations | 17.3 | 0.00 |
|  |  | Bilberry | 18.5 | 0.00 |
|  |  | (Moose observations)^2^ | 19.5 | 0.00 |
|  |  | Sex | 20.4 | 0.00 |
|  |  | Intercept only | 22.1 | 0.00 |
|  |  | (Bilberry)^2^ | 22.3 | 0.00 |
|  |  | Age | 25.3 | 0.00 |
|  |  | Proportion ants in diet | 25.8 | 0.00 |
|  |  | Proportion moose in diet | 26.3 | 0.00 |
|  |  |  |  |  |
| ***δ*^13^C** | **1-year lag** |  |  |  |
|  |  | Sex^d^ | 0 | 0.35 |
|  |  | (Moose observations)^2^ | 1.0 | 0.22 |
|  |  | Intercept only | 2.1 | 0.12 |
|  |  | (Moose harvest)^2^ | 3.5 | 0.06 |
|  |  | ln(Moose observation) | 4.0 | 0.05 |
|  |  | ln(Moose calves) | 4.0 | 0.05 |
|  |  | Moose calves | 4.3 | 0.04 |
|  |  | Moose observations | 5.45 | 0.02 |
|  |  | Age | 5.5 | 0.02 |
|  |  | (Moose calves)^2^ | 6.3 | 0.02 |
|  |  | Bilberry | 6.4 | 0.01 |
|  |  | ln(Bilberry) | 6.6 | 0.01 |
|  |  | Moose harvest | 8.0 | 0.01 |
|  |  | ln(Moose harvest) | 8.0 | 0.01 |
|  |  | (Bilberry)^2^ | 9.7 | 0.00 |
| Proportion Bilberry | No time lag |  |  |  |
|  |  | Intercept only | 0 | 1.00 |
|  |  | Bilberry + Moose calves | 19.3 | 0.00 |
|  |  |  |  |  |
|  |  | Intercept only^e^ | 0 | 0.94 |
|  |  | Year^e^ | 5.4 | 0.06 |
| Proportion moose | 1-year time lag |  |  |  |
|  |  | Bilberry + Moose harvest | 0 | 1.00 |
|  |  | Intercept only | 32.5 | 0.00 |
|  |  |  |  |  |
|  |  | Year^e^ | 0 | 1.00 |
|  |  | Intercept only^e^ | 41.0 | 0.00 |
| Variation in proportion bilberry | No time lag | Year + Demographic category | 0 | 1.00 |
|  |  | Year+ Demographic category + Year * Demographic category | 23.8 | 0.00 |
|  |  | Intercept only | 66.8 | 0.00 |
|  |  |  |  |  |
| Variation in proportion moose | No time lag | Year + Demographic category | 0 | 1.00 |
|  |  | Year+ Demographic category + Year * Demographic category | 27.5 | 0.00 |
|  |  | Intercept only | 47.7 | 0.00 |
|  |  |  |  |  |

^a^Model used for inference describing the variation in *δ*^15^N values.

^b^The model that used year as a categorical affect had the most support, but many of the model estimates have very large standard errors, likely resulting from different sample sizes across years and the composition of different reproductive categories within annual samples, thus it was not used to draw final inferences.

^c^Model used for inference describing the variation in *δ*^13^C values.

^d^The model accounting for the effect of sex in *δ*^13^C was well-supported in the data, however several components of the variance-covariance matrix were estimated at zero. This is likely because sex was modelled both in the random effects through the different reproductive categories as well as the fixed effect of sex. We chose to retain the random effect of reproduction and drop the fixed effect of sex for *δ*^13^C values.

^e^When we tested for a trend in diet proportions, we used the full data set from the years 1995–2020. Thus, these models are not comparable to the models testing the effect of resource availability, which used the subset data 2006–2020. These models also included no time lags.

Table S3. description of sample sizes used in diet analysis

| Reproductive category | number of records | number of unique bears | resampling rate | annual sampling rate |
| --- | --- | --- | --- | --- |
| Females with dependent offspring | 190 | 71 | 1-9, mean 2.7 | 3-9 |
| Solitary females | 239 | 118 | 1-8, mean 2 | 2-20 |
| All females | 429 | 146^a^ | 1-13, mean 2.9 |  |
| Solitary males | 251 | 120 | 1-9, mean 2.1 | 1-19 |

^a^ Number of unique bears in both female categories is less than the sum of both categories because the same female may be present in both categories, depending on the year.

Table S4. Model results from linear mixed modelling explaining variation in *δ*^13^C and *δ*^15^N values in brown bear hair from south-central Sweden 1995-2020 (n = 669). We tested for linear patterns as well as log-linear (ln) relationships between response and explanatory variables. We also tested whether stable isotope values responded to food availability in the same year hair was grown, or to food availability in the year prior, i.e., with a 1-year time lag; (lagged). Explanatory variables included an annual index of bilberry production (Bilberry), annual number of moose (*Alces alces*) calves produced based on hunter observations after accounting for observation effort (Calf), the annual number of moose harvested (Harvest), and bear age and sex. We used the difference in AIC*_c_* scores (ΔAIC*_c_*) and model weights (w*_i_*) to determine the most parsimonious model. Beta estimates ($\hat{\beta}$) and 95% confidence intervals (LCI, UCI) are provided for each explanatory variable in competitive models.

| Response variable | Model | K | ΔAIC*_c_* | w*_i_* | parameter | $\hat{\beta}$ | LCI | UCI |
| --- | --- | --- | --- | --- | --- | --- | --- | --- |
| Carbon isotope values (*δ*^13^C) | $Calf+ln(Bilberry)$ | 3 | 0 | 1.00 |  |  |  |  |
|  |  |  |  |  | Intercept | -0.19 | -0.57 | 0.18 |
|  |  |  |  |  | ln(Bilberry) | -0.21 | -0.30 | -0.12 |
|  |  |  |  |  | Calves | -0.25 | -0.35 | -0.14 |
|  | $Calf$ | 2 | 14.1 | 0.00 |  |  |  |  |
|  | $ln(Bilberry)$ | 2 | 15.1 | 0.00 |  |  |  |  |
|  | $Intercept$ | 1 | 22.1 | 0.00 |  |  |  |  |
| Nitrogen isotope values  (*δ*^15^N) | $Age* sex+Bilberry_{\left( lagged \right)}$ | 5 | 0 | 0.90 |  |  |  |  |
|  |  |  |  |  | intercept | -0.47 | -0.84 | -0.10 |
|  |  |  |  |  | age | 0.14 | 0.02 | 0.26 |
|  |  |  |  |  | sex (m) | 1.21 | 0.61 | 1.81 |
|  |  |  |  |  | bilberry (lagged) | -0.18 | -0.24 | -0.12 |
|  |  |  |  |  | age*male | 0.57 | 0.38 | 0.75 |
|  | $Age* sex+Bilberry_{\left( lagged \right)}+Moose Harvest_{\left( lagged \right)}$ | 6 | 4.3 | 0.10 |  |  |  |  |
|  |  |  |  |  | intercept | -0.46 | -0.81 | -0.10 |
|  |  |  |  |  | age | 0.14 | 0.02 | 0.26 |
|  |  |  |  |  | sex (m) | 1.19 | 0.61 | 1.77 |
|  |  |  |  |  | bilberry (lagged) | -0.17 | -0.23 | -0.11 |
|  |  |  |  |  | moose harvest (lagged) | -0.06 | -0.14 | 0.02 |
|  |  |  |  |  | age*male | 0.55 | 0.37 | 0.74 |
|  | $Bilberry_{\left( lagged \right)}$ | 2 | 79.2 | 0 |  |  |  |  |
|  | $Moose Harvest_{\left( lagged \right)}$ | 2 | 97.2 | 0 |  |  |  |  |
|  | $Intercept$ | 1 | 100.8 | 0 |  |  |  |  |
| Dietary proportion moose | $Bilberry_{\left( lagged \right)}+Moose harvest_{\left( lagged \right)}$ | 3 | 0 | 1.00 | Bilberry (lagged) | -0.002 | -0.003 | -0.001 |
|  |  |  |  |  | Moose harvest (lagged) | -0.002 | -0.003 | -0.001 |
|  | Intercept only | 1 | 32.5 | 0 |  |  |  |  |


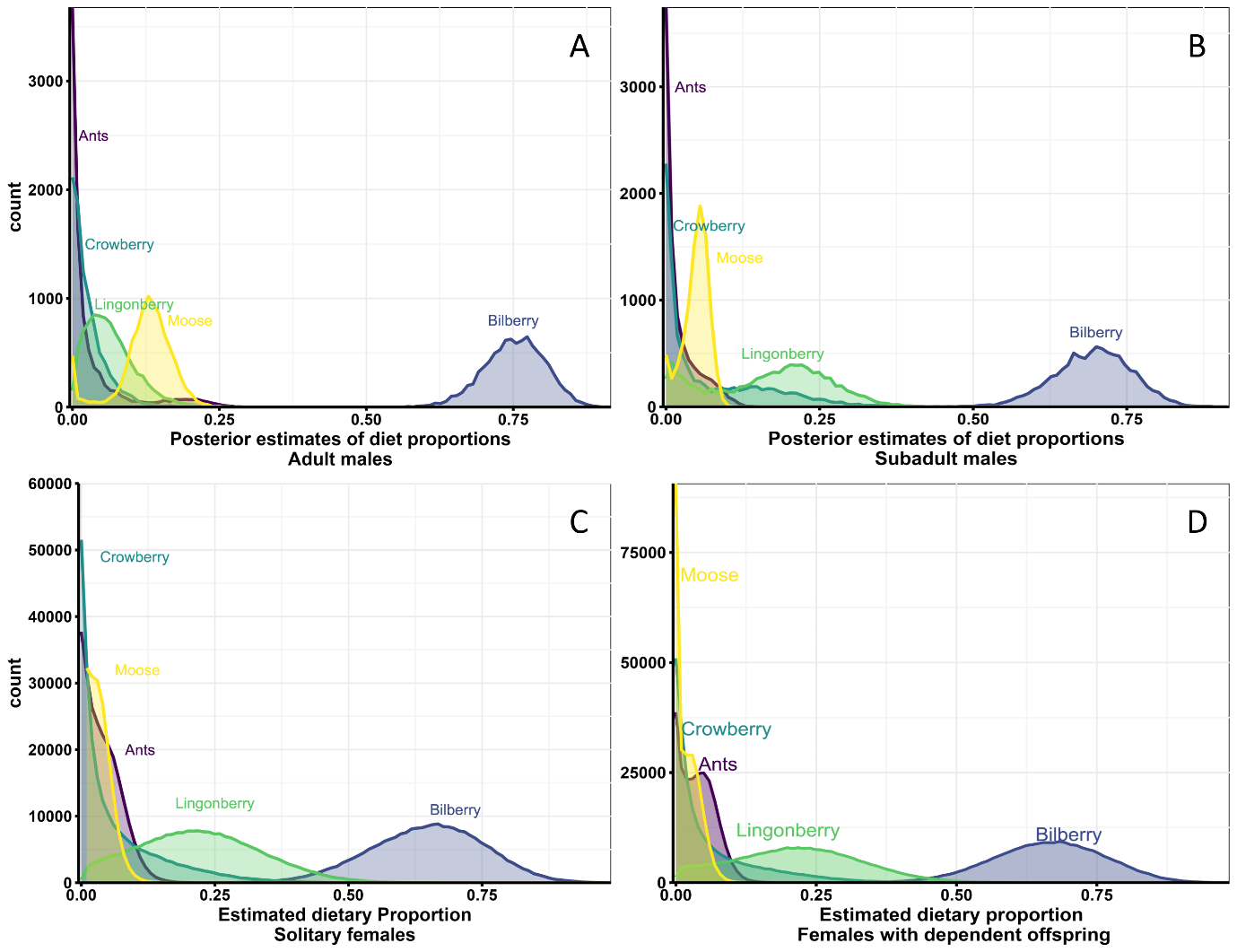


Figure S1. Posterior distribution of diet proportions for the four demographic classes of brown bears evaluated: adult solitary males (A), subadult solitary males (B), solitary females (C), and females with dependent offspring (D).


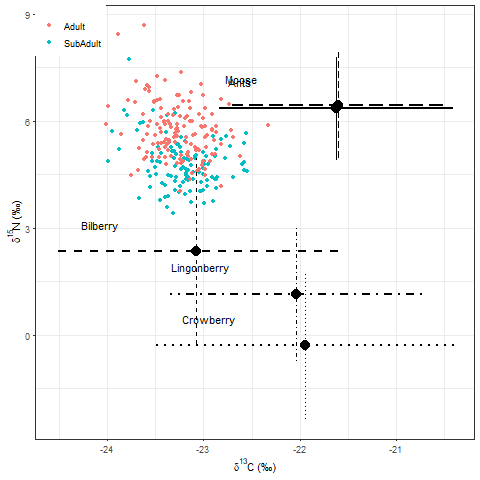


Figure S2. Mixing polygon depicting the isotopic means (large black circles) and standard errors (black lines) of foods and solitary male brown bear isotope values (small, colored circles) measured in hair and used to estimate dietary proportions for brown bears in south-central Sweden 1995–2020.


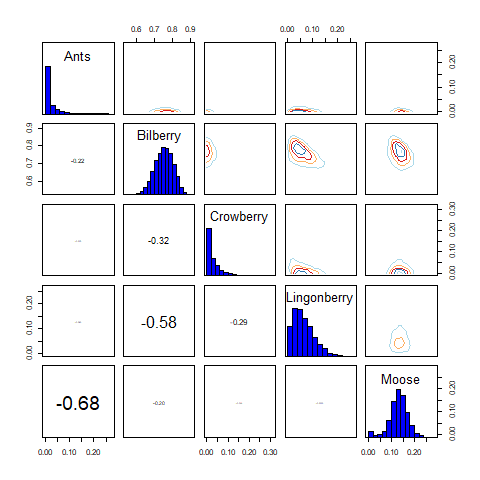


Figure S3. Plot depicting the distribution and correlations among different food sources used to estimate diet of brown bears in south-central Sweden 1995–2020.


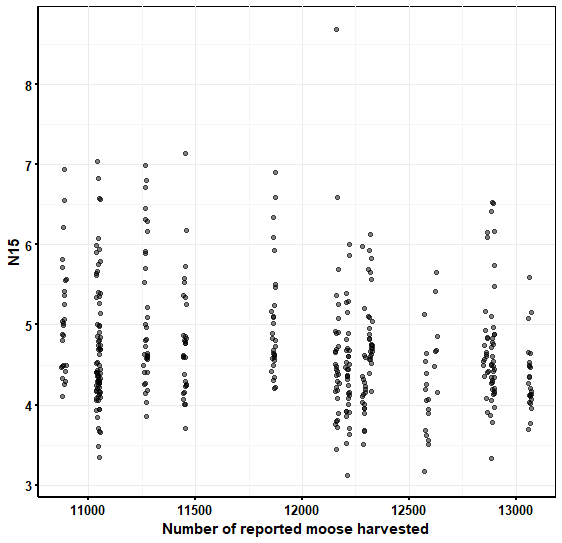


Figure S4. Observed annual number of moose harvested in the counties of Gävleborg and Dalarna, Sweden reported in the fall by civilians as part of the nation-wide statistik älgdata (<https://algdata-apps.lansstyrelsen.se>) and the *δ*^15^N values in brown bear hair from south-central Sweden 1995-2020 (n = 669).
